# Mayaro virus and dengue virus 1 and 4 natural infection in culicids from Cuiabá, state of Mato Grosso, Brazil

**DOI:** 10.1590/0074-02760150270

**Published:** 2016-01

**Authors:** Otacília Pereira Serra, Belgath Fernandes Cardoso, Ana Lúcia Maria Ribeiro, Fábio Alexandre Leal dos Santos, Renata Dezengrini Slhessarenko

**Affiliations:** 1 Universidade Federal de Mato Grosso, Centro de Ciências Básicas da Saúde I, Faculdade de Medicina, Programa de Pós-Graduação em Ciências da Saúde, Cuiabá, MT, Brasil; 2 Universidade Federal de Mato Grosso, Centro de Ciências Básicas da Saúde I, Faculdade de Medicina, Laboratório de Entomologia Médica, Cuiabá, MT, Brasil; 3 Universidade Federal de Mato Grosso, Centro de Ciências Básicas da Saúde I, Faculdade de Medicina, Laboratório de Virologia, Cuiabá, MT, Brasil

**Keywords:** dengue virus, Mayaro virus, entomological surveillance

## Abstract

This study aimed to verify the diversity of Culicidae species and their frequency of infection with flaviviruses and alphaviruses in Cuiabá, state of Mato Grosso, Brazil. Mosquitoes were captured with Nasci aspirators and hand net in 200 census tracts, identified alive at species level and pooled in one-20 (11,090 mosquitoes, 14 species). Female pools (n = 610) were subjected to multiplex seminested-reverse transcription-polymerase chain reaction (RT-PCR) for 11 flavivirus and five alphavirus. Positive pools were tested by single RT-PCR followed by nucleotide sequencing, by RT-PCR for *E1* gene [Mayaro virus (MAYV)] and by inoculation in Vero cells (MAYV) or C6/36 cells (flaviviruses). One/171 *Aedes aegypti* was positive for dengue virus (DENV)-1, 12/403 *Culex quinquefasciatus*, and four/171*Ae. aegypti* for MAYV, which was isolated from two pools containing two nonengorged females of *Ae. aegypti* and two of*Cx. quinquefasciatus*. DENV-4 was detected in 58/171 pools of *Ae. aegytpi*, 105/403 *Cx. quinquefasciatus*, two/five *Psorophora* sp., two/11 *Psorophora varipes/Psorophora albigenu*, one/one *Sabethes chloropterus*, two/five *Culex bidens/Culex interfor*, and one/one *Aedes* sp. DENV-4 was isolated from two pools containing three and 16 nonengorged *Cx. quinquefasciatus* females. Phylogenetic analysis revealed MAYV belongs to genotype L, clustering with human samples of the virus previously identified in the city. Cuiabá has biodiversity and ecosystem favourable for vector proliferation, representing a risk for arbovirus outbreaks.

Arboviruses (arthropod-borne viruses) are widely distributed, predominating in tropical areas. These viruses are maintained in nature by epidemiological cycles involving vertebrate hosts and haematophagous arthropod vectors, mainly mosquitoes, ticks, sandflies, and biting midges (Bichaud et al. 2014).

Female mosquitoes are infected by arboviruses during haematophagy in amplification hosts, such as birds, primates, and humans. Vector competence is a component of vector capacity and comprises a variety of biological and genetics factors that combined determine the ability of the invertebrate to transmit infectious agents (Beerntsen et al. 2000). After an extrinsic period of incubation of eight-14 days, a life-long persistent infection is established in the salivary glands of competent vectors (Forrester et al. 2014). Although less frequent, transovarial and venereal transmission also occur in mosquitoes (Coffey et al. 2013).

Flaviviruses transmitted by mosquitoes are divided in those with *Culex*spp as vectors, usually associated to febrile illness and encephalitis, and those transmitted by *Aedes* spp, responsible for febrile illness and haemorrhage in their hosts (Figueiredo 2000,Coffey et al. 2013). Medically important flaviviruses already reported in Brazil include yellow fever virus (YFV), dengue virus (DENV) serotypes, Saint Louis encephalitis virus (SLEV), Ilhéus virus (ILHV), Rocio virus (ROCV), Bussuquara virus (BSQV), Cacipacoré virus (CPCV), and recently, West Nile virus (WNV) and Zika virus (ZIKV) (Figueiredo 2000, Batista et al. 2011, WHO 2014). Evidences of flaviviruses circulation in Brazil have also been reported in birds (WNV, SLEV, ROCV, and ILHV), rodents [SLEV and Iguape virus (IGUV)] (Rodrigues et al. 2010,Batista et al. 2011), equines (WNV, SLEV, ROCV, ILHV and CPCV) (Pauvolid-Corrêa 2008, Pauvolid-Corrêa et al. 2014, Silva et al. 2014), and in vector species (except for WNV) (Cardoso et al. 2010, Figueiredo et al. 2010).

The genus *Alphavirus*, family Togaviridae*,* include the eastern (EEEV), western (WEEV), and Venezuelan (VEEV) equine encephalitis viruses (Alice 1951, Iversson et al. 1990), Mayaro virus (MAYV) (Mourão et al. 2012, Zuchi et al. 2014), and Chikungunya virus (CHIKV) (Bronzoni et al. 2005,Formenti 2015). EEEV, VEEV, and WEEV were reported in equines (Aguiar et al. 2008, Pauvolid-Corrêa 2008, Melo et al. 2012) and several alphaviruses (EEEV, VEEV, WEEV, MAYV, Pixuna virus) were described in arthropod vectors (Shope et al. 1964, Estep et al. 2011, Long et al. 2011).

Surveillance studies involving entomology and virology are important tools for monitoring the mosquito fauna and determining intervention strategies to control and prevent arbovirus epidemics (Regis et al. 2009). Control measures usually target *Aedes* (*Stegomyia*) populations in urban areas. Other Culicidae species, also important for arbovirus transmission, are being neglected (Cardoso et al. 2010).

The state of Mato Grosso (MT), located in Central-West Brazil, presents ecological and climatic conditions favourable to arbovirus circulation. The state is composed of the Amazon, Pantanal, and *Cerrado* biomes. Cuiabá is the capital of MT and a significant number of confirmed or suspected dengue cases are reported annually in the city (Acendino 2013). Studies involving patients with acute febrile illness during a large dengue outbreak demonstrated the hyperendemicity of DENV and the circulation of SLEV and MAYV in Cuiabá during 2012 (Zuchi et al. 2014, Heinen et al. 2015a, b). However, until the present, studies to evaluate the genetic diversity and frequency of these viruses are lacking. Toward these ends, the present study was aimed at verifying the diversity of Culicidae species and their frequency of infection by flaviviruses and alphaviruses in Cuiabá. Mosquitoes present a life-long persistent infection and are a common host for several arboviruses. Moreover, these invertebrates represent an important tool for determining the epidemiological status of arboviruses in a given area.

## MATERIALS AND METHODS

*Study area and Culicidae capture* - Cuiabá is located in the south-central region of the state, between the coordinates 15º35’56’’S 56º06’01’’W, comprising 3,495,424 Km^2^ of surface area and 575,480 inhabitants. The climate is semihumid tropical with mean temperature ranging from 21.4-32.8ºC, characterised by a dry season from April-September and a rainy season between October-March (IBGE 2011).

Cuiabá is administratively divided into four regions (north, south, central-east and midwest) and 173 neighbourhoods. The neighbourhoods are further subdivided into 804 census tracts (IBGE 2011), based on demographic density (each census tract represents 250-350 residencies) among which 200 census tracts were randomly selected and located using GPS for adult Culicidae collection between January-April, 2013. Three sites were sampled for at least 30 min within each selected unit between 01:00 p.m.-05:00 p.m., using Nasci aspirators (Horst Armadilhas, Brazil) and hand net. The adult specimens were transferred immediately to entomological recipients using manual suction tube (Castro catcher) and transported with adequate humidity and temperature conditions provided with access to 20% glucose solution.

At the laboratory, mosquitoes were identified alive, in a dormant state (4 min at 4ºC), using a dichotomous key (Forattini 2002) within 12-24 h. Alternatively, a nested polymerase chain reaction (PCR) for *Culex quinquefasciatus* identification (Smith & Fonseca 2004) was performed in 193 pools of*Culex* sp. after DNA extraction, since morphological identification of the species was not possible in these pools. They were grouped according to date, collection site, species, and gender in pools of one-20 specimens, and stored at -80ºC immediately.

*Total RNA and DNA extraction* - Specimens were macerated in 800 µL of phosphate-buffered saline and centrifuged at 5,500 *g* for 4 min at 4ºC. Total RNA was extracted from 400 µL of the supernatant with Trizol reagent (Invitrogen, USA). The pellet obtained during RNA extraction of*Culex* sp. pools (n = 193) was subjected to total DNA extraction. Procedures followed the manufacturer’s instructions.

*Multiplex seminested-reverse transcription-PCR (RT-PCR) for Flavivirus and Alphavirus genera and nucleotide sequencing* - cDNAs were obtained from the samples by RT of the extracted RNA (9.1 µL) with genera specific primers [FG2 (15 µM) for flaviviruses, cM3W (100 µM) for alphaviruses] (Bronzoni et al. 2005), 100 U of reverse transcriptase (Superscript III; Invitrogen), 20 U of RNAse inhibitor (RNAse OUT; Invitrogen), DNTP mix, buffer, dithiothreitol, and temperature conditions following the manufacturer’s instructions.

cDNA (8 µL) was subjected to duplex-PCR for a NS5 region (958 bp) of*Flavivirus* and a nsP1 region (434 bp) of*Alphavirus* genus, followed by three species-specific multiplex-seminested-PCR reactions: flaviviruses 1 (DENV-1, 2, 3, YFV, and SLEV), flaviviruses 2 (DENV-4, ILHV, BSQV, IGUV, ROCV, and WNV), and alphaviruses (MAYV, Aura virus, EEEV, WEEV, and VEEV), as previously described (Bronzoni et al. 2005, Cruz et al. 2015).

Positive samples were subjected to single RT-PCR with the same forward and reverse primers for the respective flaviviruses or alphaviruses species identified in the multiplex. The PCR product of these single reactions was purified with polyethylene glycol precipitation protocol, subjected to nucleotide sequencing (3500 genetic analyser; Applied Biossistems, USA), analysed using Geneious R6 v.6.0 and compared through BLASTn with reference sequences available in GenBank [National Center for Biotechnology Information (NCBI)]. Precautions to avoid cross-contamination were carefully undertaken during the entire procedure.

*RT-PCR for a region of the envelope gene 1 (E1) of alphaviruses* - A region of the *E1* of MAYV-positive pools was amplified as described by Powers et al. (2001) with few modifications. Briefly, total RNA was reverse transcribed with the primer T_25_V-Mlu (50 µM), 2.5 mM MgCL_2_, 100 U of reverse transcriptase (GoScript; Promega, USA), 20 U of RNAse inhibitor (RNAse OUT, Invitrogen) and 1x buffer according to cycling conditions described by the manufacturer. PCR amplification was performed with 6 µL of cDNA, 50 µM of the primer α10247A, 10 mM of dNTP Mix, 2 mM MgCL_2_, 1x buffer, 1 U of DNA polymerase (GoTaq Hot Start; Promega), and ultrapure water to 50 µL reaction volume. Cycling conditions were used as described by the authors, except for the annealing temperature (45ºC instead of 49ºC). The PCR bands (1.3 kb) were excised from the agarose gel, purified with nucleospin kit (Macherey-Nagel, Germany), and sequenced as previously described.

*Inoculation of positive pools in cell culture* - For virus isolation, the supernatants of MAYV-positive pools were inoculated at 1:10 dilution in Vero cells (ATCC CCL-81) and the pool positive for DENV-1, 10 for DENV-4, and one for SLEV in C6/36 cells (ATCC CLR-1660) cultivated in 24-well polystyrene plates. After 2h of incubation, the inoculum was removed and the monolayers were washed with RPMI-1640 medium (Vero cells) or L-15 medium (C6/36 cells) containing antibiotics. Culture medium (RPMI-1640 or L-15 with 5% foetal bovine serum) was replaced, the plates maintained at 37ºC (Vero cells) or 28ºC (C6/36 cells) in 5% CO_2_incubators, and the monolayers monitored for seven days by examination through inverted microscope. The supernatant was stored at -80ºC and the monolayers subjected to total RNA extraction for single-RT-PCR as described above. Three passages in cell culture were performed for each pool.

*Data analysis* - Data according to census tract, date of capture, species, gender, and number of specimens were compiled using Microsoft Excel 2013. Minimum infection rate (MIR) was calculated using the formula [(number of positive pools/total specimens of the species tested) x 1,000] (Chow et al. 1998) (Table) and presented with confidence interval of 95% (Epidata Analysis, 2006-2010). Geospatial data were plotted in maps (ArcMap, ESri ArcGIS).

**TABLE:** Natural infection frequency and minimum infection rate (MIR) of adult Culicidae female pools positive for dengue virus 4 (DENV-4) and Mayaro virus (MAYV) by administrative region of Cuiabá, state of Mato Grosso, Brazil, 2013

Species	Neighbourhood	Collected pools (n)	Females (n)	Positive pools (n)	MIR ( ± CI)	
					DENV-4	MAYV
North						
*Aedes aegypti*	Paiaguás, Centro Político Administrativo, Morada da Serra, Primeiro de Março, Três Barras	29	129	4	31.01 (± 1.7)	-
*Aedes* sp.	Jardim Vitória	1	1	1	1,000	-
*Culex bidens*/	Três Barras	1	1	0	-	-
*Culex interfor*						
*Culex quinquefasciatus*	Paiaguás, Centro Político Administrativo, Morada da Serra, Três Barras, Primeiro de Março	83	768	12	15.42 (± 3.92)	-
*Culex spinosus*	Morada da Serra	1	1	0	-	-
*Psorophora* sp.	Jardim Vitória	1	1	0	-	-
*Psorophora varipes*/	Centro Político Administrativo	2	4	0	-	-
*Psorophora albigenu*						
South						
*Ae. aegypti*	Jardim Petrópolis, Jardim Paulista, Campo Velho, Pedra 90, Tijucal, São João Del Rei, Altos do Coxipó, Coxipó, Jardim das Palmeiras, Santa Laura, São Sebastião, Osmar Cabral, Parque Geórgia, Residencial Coxipó,São Francisco, Nova Esperança, Jardim Industriário, Jardim Comodoro, Parque Cuiabá, Parque Atalaia, Jardim Florianópolis	45	220	27^*a*^	113.64 (± 1.13)	9.09 (± 3.97)
*Cx. bidens*/ *Cx. interfor*	Pedra 90	1	1	0	-	-
*Cx. quinquefasciatus*	Campo Velho, Pedra 90, Tijucal, São João Del Rei, Altos do Coxipó, Coxipó, Jardim Fortaleza, Santa Laura, Osmar Cabral, Bela Marina, Jardim Gramado, Residencial Coxipó, São Francisco, Jardim Industriário, Parque Atalaia, Parque Cuiabá, Jardim Comodoro, Nossa Senhora Aparecida, Jardim Petrópolis, Jardim Paulista, São Sebastião, Parque Geórgia, Nova Esperança, Jardim Passaredo, Jardim Presidente	149	1,409	58	39.61 (± 2.9)	1.39 (± 17.6)
*Psorophora* sp.	Osmar Cabral	1	1	1	1,000	-
*Ps. varipes*/	Jardim Presidente	1	1	1	1,000	-
*Ps. albigenu*						
*Sabethes chloropterus*	Parque Cuiabá	1	1	1	1,000	-
Central-east						
*Ae. aegypti*	Araés, Lixeira, Bosque da Saúde, Poção, Dom Aquino, Areão, Jardim das Américas, Pedregal, Terra Nova, Carumbé, Bela Vista, Jardim Itália, Santa Cruz, UFMT, Boa Esperança, Jardim Leblon, Praeirinho, Terceiro, Grande Terceiro, Recanto dos Pássaros, Jardim Imperial, Jardim Petrópolis, Jardim Califórnia, Campo Velho, Campo Verde, Jardim Universitário, Altos do Coxipó, Residencial Itamaraty, Planalto, Novo Horizonte	54	457	25^*b*^	46.12 (± 2.48)	4.23 (± 9.2)
*Ae. albopictus*	Novo Mato Grosso	1	1	0	-	-
*Cx. bidens*/	Bosque da Saúde, Bela Vista	2	2	2	1,000	-
*Cx. interfor*						
Central-east						
*Cx. quinquefasciatus*	Araés, Lixeira, Bosque da Saúde, Poção, Terceiro, Dom Aquino, Areão, Jardim das Américas, Pedregal, Terra Nova, Carumbé, Bela Vista, Jardim Itália, Santa Cruz, UFMT, Boa Esperança, Jardim Leblon, Grande Terceiro, Terceiro, Praeirinho, Recanto dos Pássaros, Jardim Imperial, Campo Velho, Campo Verde, Jardim Universitário, Jardim dos Ipês, Altos do Coxipó, Planalto, Jardim Eldorado, Novo Horizonte, Jardim Petrópolis, Residencial Itamaraty, Novo Mato Grosso	114	628	31^*a*^	38.21 (± 1.74 )	16 (± 2.7)
*Limatus* sp.	UFMT, Recanto dos Pássaros	2	3	0	-	-
*Psorophora* sp.	Araés, UFMT	2	2	1	500	-
*Ps. varipes*/	Araés, Bosque da Saúde, UFMT, Novo Horizonte, Dom Aquino	5	5	1	200	-
*Ps. albigenu*						
Midwest						
*Ae. aegypti*	Barra do Pari, Santa Isabel, Coophamil, Novo Terceiro, Porto, Cidade Alta, Goiabeiras, Jardim Cuiabá, Duque de Caxias, Novo Colorado, Jardim Mariana, Quilombo, Despraiado, Santa Marta, Araés, Lixeira, Bosque da Saúde, Alvorada, Paiaguás, Centro Sul, Centro Norte	43	281	6	21.35 (± 1.73)	-
*Cx. bidens*/	Jardim Mariana	1	3	0	-	-
*Cx. interfor*						
*Cx. quinquefasciatus*	Barra do Pari, Santa Isabel, Coophamil, Cidade Alta, Porto, Goiabeiras, Duque de Caxias, Jardim Cuiabá, Novo Colorado, Jardim Mariana, Quilombo, Despraiado, Santa Marta, Araés, Lixeira, Alvorada, Bosque da Saúde, Paiaguás, Santa Isabel, Centro Sul, Centro Norte	57	620	12^*c*^	19.35 (± 5.45)	-
*Galindomyia* sp.	Barra do Pari	1	1	0	-	-
*Limatus* sp.	Barra do Pari, Quilombo, Centro Norte	4	4	0	-	-
*Mansonia wilsoni*	Coophamil, Despraiado	2	4	0	-	-
*Psorophora ciliata*	Santa Marta	1	1	0	-	-
*Psorophora* sp.	Araés, Alvorada	1	1	0	-	-
*Ps. varipes*/ *Ps. albigenu*	Quilombo, Santa Marta, Araés, Bosque da Saúde, Alvorada	3	4	0	-	-
*Uranotaenia* sp.	Despraiado	1	1	0	-	-

*a*: MAYV was isolated from one pool containing two females of *Ae. aegypti* captured in São Sebastião and from another with two females of *Cx. quinquefasciatus* collected at Bela Vista neighbourhood, both positive only for MAYV by reverse transcription-polymerase chain reaction; *b*: one of these pools containing 18 females of *Ae. aegypti* captured at Bela Vista neighbourhood was positive for DENV-1; *c*: DENV-4 was isolated from one pool containing three females of *Cx. quinquefasciatus* captured at Porto neighbourhood and from another with 16 females of the same species captured in Pedra 90 neighbourhood; CI: confidence interval; UFMT: Universidade Federal de Mato Grosso.

*Phylogenetic analysis* - A neighbour-joining phylogenetic three was generated with the nucleotide sequences of a region of *NsP1* gene amplified from MAYV-positive pools with Tamura-Nei distance model and 1,000 bootstrap (Geneious software 6.0). Reference sequences, previously classified in genotypes L and D, were retrieved from GenBank (PubMed; NCBI) for comparison. Outgroups included Trocara virus, EEEV, and CHIKV.

*Accessions of sequences deposited in GenBank* - Nucleotide sequences obtained in this study were deposited at GenBank with the accessions DENV-1 (KP710881), DENV-4 (KP694224, KP694225, KP710879, KP710880, and KP742343), and MAYV (KP710882-KP710893, KP742341, KP742342, and KP954632).

## RESULTS

*Mosquitoes collected in Cuiabá* - Between January-April 2013, 11,090 adult Culicidae specimens were collected in Cuiabá, including 6,534 (58.9%) males and 4,556 (41.1%) females. This resulted in 1,419 pools, of which 610 were comprised of females, 114 collected in the midwest region, 180 in the central-east, 118 from the north, and 198 from the south of the city (Table).

Species belonging to *Culex* (8,759 specimens; 79%) and*Aedes* (2,294 specimens; 20.7%) genera were the most abundant. Eight genera of Culicidae were represented in the collections, comprising 14 species: *Ae*. *aegypti* (n = 2,291),*Ae*des *albopictus* (n = 2), *Aedes* sp. (n = 1), *Culex bidens* or *Culex interfor* (n = 7), *Cx. quinquefasciatus* (n = 8,751), *Culex spinosus*(n = 1), *Galindomyia* sp*.* (n = 1),*Limatus* sp*.* (n = 8), *Mansonia wilsoni*(n = 4), *Psorophora* (*Psorophora*) sp*.* (n = 7), *Psorophora ciliata* (n = 1), *Psorophora varipes* or *Psorophora albigenu*(n = 14), *Sabethes chloropterus* (n = 1), and*Uranotaenia* sp*.* (n = 1).

*Frequency of infection by arboviruses in adult female mosquitoes* - Since only females are haematophagous and due to the large number of specimens collected in the experiment, only the 610 female pools were tested for arboviruses. Of these, 28% (n = 171) were positive for flaviviruses and 2.6% (n = 16) for alphaviruses (Table). Pools of adult females positive for more than one arbovirus represented 0.1%, with three of these five pools with dual infection by DENV and MAYV being composed of only one female without signs of engorgement.

**Fig. 1:**
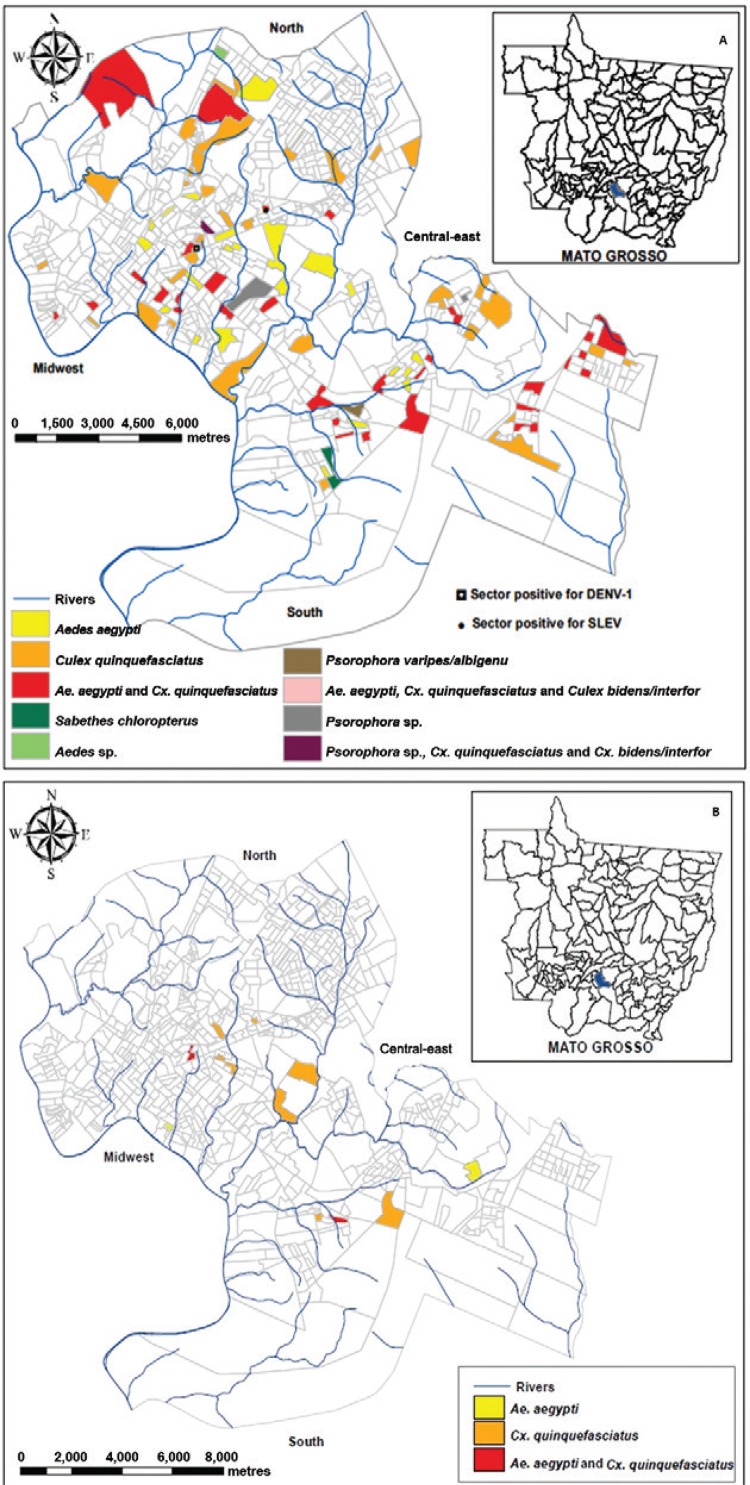
: distribution of adult culicid female pools positive for dengue virus serotype 1 (DENV-1), DENV-4, and for Saint Louis encephalitis virus (SLEV) (1A) and Mayaro virus (1B) in urban sectors of the city of Cuiabá, state of Mato Grosso, Brazil.

One pool obtained at Bela Vista neighbourhood, captured in February 2013 in the central-east region of Cuiabá containing 18 nonengorged specimens of *Ae. aegypti,* was positive for DENV-1 (MIR = 0.92 ± 1.7) (KP710881), DENV-4, and MAYV (KP710882) (Fig. 1). Its nucleotide sequence presented, respectively, 99% identity with the DENV-1 HNRG27213 (KC692513.1), genotype V, American/African lineage strain, obtained from humans in 2010 in Argentina, and 99% with the MAYV sequence MAY_BR/MT_CBA_230/2012 obtained from humans in 2012, in Cuiabá (KJ879253.1). One pool obtained in other collection site in the same neighbourhood, containing one female of *Cx. quinquefasciatus* captured on the same day, was positive for SLEV. The nucleotide sequence (KJ801827) presented 99% of identity with the SLEV previously identified in humans with acute febrile illness in MT during a severe dengue outbreak, belonging to genotype VA (Heinen et al. 2015b) (Fig. 1A).

DENV-4 was detected in 171 (28%) pools: 58/171 (33.9%) of *Ae. aegypti,*one/one (100%) of *Aedes* spp, 105/403 (26%) of *Cx. quinquefasciatus,* two/five (40%) of *Cx*.*bidens*/*interfor,* two/five (40%) of*Psorophora* sp., two/11 (18.2%) of *Ps. varipes/albigenu*, and one/one (100%) of *Sa. chloropterus* (Fig. 1A, Table). Among these pools, 44 *Culex* spp, two *Psorophora*spp, and the *Sa. chloropterus* pool contained engorged females. Nucleotide sequencing demonstrated the identity to be 99% to DENV-4 isolates of genotype II from Manaus, state of Amazonas (H780563, JQ513343.1, and H780556, JQ513342.1). DENV-4 was isolated from two pools of *Cx. quinquefasciatus* containing 16 (#806, 1st passage) and three (#329, 1st passage) nonengorged females.

Pools of *Cx. quinquefasciatus* (12/403; 3%) and *Ae. aegypti*(4/171; 2.3%) obtained in 12 census tracts were positive for MAYV (Fig. 1B, Table), among which five were also positive to DENV-4. Two pools positive only to MAYV containing two nonengorged females, one of *Cx. quinquefasciatus* (#489, 3rd passage; KP742341) and one of *Ae. aegypti* (#958, 1st passage; KP742342), presented cytopathic effect after inoculation in Vero cells. Virus isolation was confirmed through single-RT-PCR and nucleotide sequencing.

**Fig. 2:**
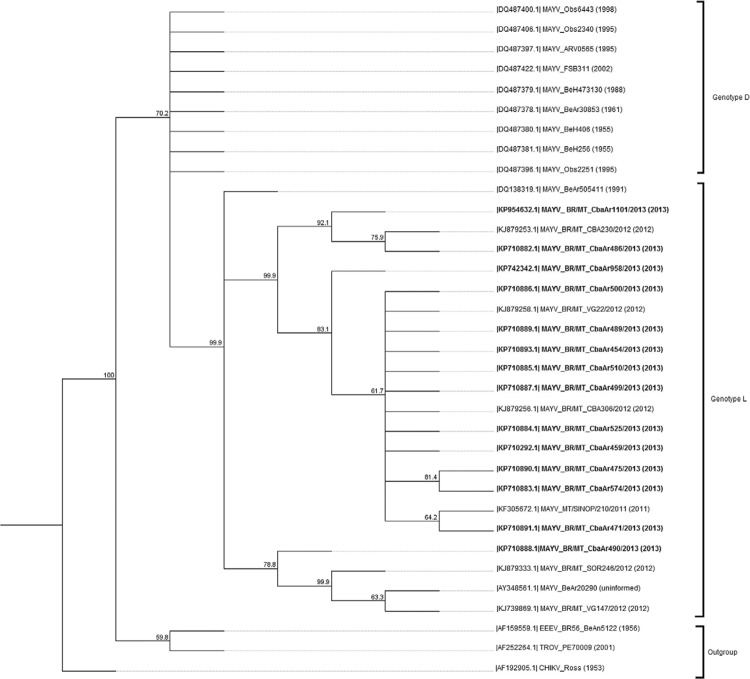
: phylogenetic analysis of Mayaro virus (MAYV) *nsP1*gene partial sequences identified in culicids captured in the urban area of Cuiabá, state of Mato Grosso, Brazil, in comparison with reference sequences obtained through GenBank (National Center for Biotechnology Information). Tree was generated by neighbour-joining method and Tamura-Nei distance method, 1,000 bootstrap replicates. Outgroup consisted of Trocara virus (TROV), eastern equine encephalitis virus (EEEV), and Chikungunya virus (CHIKV). Sequences identified in this study are in bold.

Unspecific amplification of mosquito sequences was identified within the 1.3 kb amplicon obtained through the *E1* gene RT-PCR in MAYV positive pools. Phylogenetic analysis of the nsP1 partial sequences obtained from these pools revealed a similarity with sequences of the virus obtained from human samples in MT. These sequences presented a high similarity with other sequences of MAYV belonging to genotype L from *Ixodes* spp, *Haemagogus janthinomys*and humans in the state of Pará (PA) (Fig. 2).

Among the four administrative regions of Cuiabá, the south presented 44.4% (88/198) pools positive for arboviruses, followed by central-east with 33.8% (61/180), north with 14.4% (17/118), and midwest with 15.8% (18/114). No statistical difference was observed for sylvatic species (*Psorophora*,*Sabethes*, *Limatus*, *Mansonia*, and *Uranotaenia*) distribution between the four administrative regions (Table).

## DISCUSSION

This is the first study to investigate the diversity of Culicidae species and their frequency of infection by medically important flaviviruses and alphaviruses in Cuiabá. *Cx. quinquefasciatus* and *Ae. aegypti*occurrence has been previously documented in Cuiabá (Campos & Andrade 2003, Carvalho-Leandro et al. 2010). In 2012, DENV hyperendemicity and SLEV and MAYV circulation were also reported in the city (Zuchi et al. 2014, Heinen et al. 2015a, b). In the same year, the frequency of transovarial infection of *Ae. aegypti* by DENV-4 was 10.5% in a region of Cuiabá with a high incidence of dengue (Cruz et al. 2015). However, studies involving adult Culicidae and other arboviruses were lacking in this area. Generally, mosquitoes infected by arboviruses are detected for up to six weeks prior to the beginning of an outbreak (Chow et al. 1998). Moreover, infected mosquitoes carry the virus for their entire life, presenting as an epidemiological indicator of arboviruses circulation in a given area.

*Cx*. *quinquefasciatus*, the most abundant species, was present in 92% of the census tracts and was positive for DENV-4, SLEV, and MAYV (Fig. 1, Table). The prevalence of this species might have resulted from the competitive success for breeding sites and blood meal sources in comparison to other culicids. This species is a competent vector for WNV, SLEV, Oropouche virus, and MAYV (Hoch et al. 1987, Segura & Castro 2007, Abad-Franch et al. 2012).

Data concerning SLEV circulation in Brazil are probably underestimated. Human cases were reported in the Amazon Region, in the city of São Paulo (Rocco et al. 2005, Mondini et al. 2007), and in Cuiabá (Heinen et al. 2015b). One pool containing a nonengorged female of *Cx. quinquefasciatus* from midwest Cuiabá was positive for SLEV subgenotype V-A during this study; the nucleotide sequence of which presented a high similarity to the SLEV detected in humans in this same area in 2012 (Heinen et al. 2015b).

MAYV is endemic in the Amazon Region, where is maintained in sylvatic cycles involving *Ha. janthinomys* as the main vector and birds and primates as amplifying hosts. Human infections are generally incidental, due to occupational exposure (Pinheiro & Leduc 1998). An urban cycle of transmission has been proposed for MAYV in Manaus (Mourão et al. 2012) and Cuiabá (Zuchi et al. 2014). In this study, viral isolation was achieved from two pools of *Cx. quinquefasciatus* and*Ae. aegypti,* which were only positive to MAYV, with no signs of engorgement. Isolation of MAYV from other Culicidae species such as*Aedes*, *Culex*,*Psorophora*, *Sabethes*, and *Haemagogus*spp has been reported (Pinheiro & Leduc 1998, Long et al. 2011). Vector competence of *Cx. quinquefasciatus* and *Ae. aegypti*for MAYV transmission has been demonstrated experimentally (Long et al. 2011, Abad-Franch et al. 2012). The importance of these species as MAYV vectors is based on their urban and anthropophilic habits and their wide geographic distribution, favouring the contact with humans and, therefore, viral transmission and urbanisation of MAYV. The positivity of these species to MAYV in the present study might have resulted from natural infection in the mosquito, since most of the positive pools were not engorged and therefore the virus detected was not acquired from the blood. Additional studies are necessary to elucidate the involvement of these mosquito species in the epidemiological cycle of MAYV.

Nucleotide sequences of MAYV obtained from *Cx. quinquefasciatus*showed 98-100% identity with sequences of the virus obtained from humans in Cuiabá (KJ879256.1; KJ879257.1) and in the city of Várzea Grande, MT (KJ879258.1). In a similar vein, sequences obtained from *Ae. aegypti* showed 99-100% of identity with virus sequences obtained from humans in Cuiabá (KJ879253.1; KJ869256.1) during 2012, indicating the same virus was identified in humans and in mosquitoes in MT (Zuchi et al. 2014). The patients positive for MAYV in Cuiabá in 2012 were urban residents and denied recent travel to rural or sylvatic areas. Associations to sex and occupation were not identified (Forattini 2002). Taken together, these findings corroborate the occurrence of urban transmission in Cuiabá.

Although *E2*/*E1* structural sequences of alphaviruses are more appropriate for phylogenetic analysis, a similarity in the clustering among*E2*/*E1* and nsP1 sequences has been described (Vieira et al. 2015). This finding was also observed in this study. MAYV nucleotide sequences formed a monophyletic group separated in clusters comprising genotypes L and D. The genotype D is composed of several isolates from South America and genotype L of strains from northern and central regions of Brazil (Powers et al. 2006, Vieira et al. 2015).

All mosquitoes and human sequences of nsP1 from MT grouped within the genotype L of MAYV in the same cluster and are in close proximity to sequences obtained from*Ixodes* spp and humans in PA. These results indicate that the same virus might be circulating in mosquitoes and humans in this region. Vieira et al. (2015) suggested that MAYV isolates consist of a sympatric group in MT.

*Ae. aegypti* females were the second most frequent specimens, captured in 165/200 census tracts of Cuiabá. This species is highly anthropophilic, extremely adaptable to diverse urban environmental conditions, and it is widely distributed in Brazil. Monitoring the distribution and frequency of infection of this species is important in identifying the hot spots for arbovirus transmission in the city (Regis et al. 2009). *Ae. aegypti* has also been implicated in the transmission cycle of other arboviruses already described in Brazil, such as YFV, ROCV, ILHV, BSQV, ZIKV, and CHIKV (Schatzmayr 2001, Vega-Rúa et al. 2014).

DENV-1, 4 were identified among culicids in Cuiabá. The increased incidence of DENV-4 in humans in the metropolitan area of Cuiabá during 2011-2012 (Heinen et al. 2015a) was accompanied by a significant frequency of this serotype in mosquitoes during 2013. Among 171 pools positive for DENV-4, 58/171 (9%) were of *Ae. aegypti. Culex*, *Psorophora*, and *Sabethes* spp positivity to DENV-4 may have resulted from the presence of viraemic blood in the abdomen of engorged females, due to haematophagy in humans or from natural infection of these specimens. Ng et al. (2011) also reported the detection of viruses in mosquitoes arising from the alimentary source. Natural infection of different mosquito species with arboviruses is commonly described; although there is no confirmed association with vector competence for DENV serotypes for these species, i.e., participation in the transmission cycle. Moreover, *Cx. quinquefasciatus* naturally infected by DENV-2 after a blood meal on viraemic host was already described (Luo 1993) and *Haemagogus leucocelenus* positivity for DENV-1 in Bahia, where a sylvatic cycle of transmission was suggested, represents a possible adaptation of urban viruses in sylvatic mosquitoes and their maintenance in enzootic cycles (Figueiredo et al. 2010). In Brazil, natural infection of *Psorophora* spp by Maguari virus, ILHV, MAYV, YFV, and ROCV (de Souza-Lopes et al. 1981), and of *Sa. chloropterus* by SLEV*,*an important YFV vector, were reported previously (Svoboda et al. 2014).

Most of the collected mosquitoes and positive pools are from the south, followed by central-east, north and midwest regions of the city. These regions also present socioeconomic conditions favouring the proliferation of mosquito populations. The south and central-east regions presented elevated MIR relative to other regions. In 2013 alone, 3,750 dengue cases were reported in Cuiabá (SES-MT 2014). The neighbourhoods with higher dengue incidence are part of the administrative regions with a high number of positive pools. Higher rates of *Ae. aegypti* infection correlate with increased possibility of DENV transmission and are directly linked to human infections, since most adult females become infected after biting an infected host (Chow et al. 1998, Urdaneta et al. 2005, Zeidler et al. 2008).

Dengue hyperendemicity was previously described in Manaus, in the city of Rio de Janeiro, and Cuiabá (Nogueira & Eppinghaus 2011, Bastos et al. 2012, Heinen et al. 2015a). The co-circulation of more than one DENV serotype associated to susceptibility of human populations to the serotypes and density of *Ae. aegypti* populations are factors contributing to the occurrence of epidemics (Guedes 2012). For these reasons, understanding vector population characteristics is essential to establish appropriate vector control measures and, consequently, checking arboviruses dissemination.

The high mosquito population density identified in the study may explain dual positivity for MAYV and DENV-4 detected in five pools. Since most of the pools are composed of more than one specimen, probably different females were infected by different arboviruses, although co-infection could also occur and one or both viruses could also originate from haematophagy of a viraemic hosts in the pools with engorged females. Co-infections by DENV and CHIKV in *Ae*.*albopictus* pools have been described from Africa, raising the possibility of human dual infection through one single mosquito bite. Experimentally, co-infection and super-infection of this species by both viruses have been demonstrated (Vazeille et al. 2010).

Cuiabá represents a heterogeneous environmental setting. The uncontrolled demographic growth, precarious sanitary conditions, and of infrastructure observed in several neighbourhoods, associated to the presence of growing areas, parks, streams, and rivers inside the urban perimeter, may explain the presence of sylvatic culicid species in the study. Permanent preservation areas (extensive areas with native sylvatic woods in the urban perimeter protected by law) and parks also allow the existence of birds and primates that are important amplifying hosts for several arboviruses (Navarro-Silva et al. 2004).

The knowledge of culicid species diversity and their frequency of infection by arboviruses in regions where epidemics are frequent are important in predicting which arboviruses may circulate in humans or disseminate after their introduction. These observations highlight the necessity to deepen our understanding about the epidemiological cycle and molecular evolution of these viruses in MT, as well as to improve entomological surveillance measures.

## References

[B1] Abad-Franch F, Grimmer GH, Paula VS, Figueiredo LTM, Braga WSM, Luz SLB (2012). Mayaro virus infection in Amazonia: a multimodel
inference approach to risk factor assessment. PLoS Negl Trop Dis.

[B2] Acendino A (2013). Estado divulga dados de dengue de 1 de janeiro a 28 de novembro
de 2013.

[B3] Aguiar DM, Cavalcante GT, Lara MCCSH, Villalobos EMC, Cunha EMS, Okuda LH, Stéfano E, Nassar AFC, Souza GO, Vasconcellos SA, Labruna MB, Camargo LMA, Gennari SM (2008). Prevalência de anticorpos contra agentes virais e
bacterianos em equideos do município de Monte Negro, Rondônia, Amazônia ocidental
brasileira. Braz J Vet Res Anim Sci.

[B4] Alice FJ (1951). Encefalomielite equina na Bahia - estudo de três
amostras isoladas. Rev Bras Biol.

[B5] Bastos MS, Figueiredo RMP, Ramasawmy R, Itapirema E, Gimaque JBL, Santos LO, Figueiredo LTM, Mourão MPG (2012). Simultaneous circulation of all four dengue serotypes in
Manaus, state of Amazonas, Brazil in 2011. Rev Soc Bras Med Trop.

[B6] Batista WC, Tavares GSB, Vieira DS, Honda ER, Pereira SS, Tada MS (2011). Notification of the first isolation of Cacipacore virus
in a human in the state of Rondônia, Brazil. Rev Soc Bras Med Trop.

[B7] Beerntsen BT, James AA, Christensen BM (2000). Genetics of mosquito vector competence. Microbiol Mol Biol Rev.

[B8] Bichaud L, Lamballerie X, Alkan C, Izri A, Gould EA, Charrel RN (2014). Arthropods as a source of new RNA
viruses. Microb Pathog.

[B9] Bronzoni RVM, Baleotti FG, Nogueira RMR, Nunes M, Figueiredo LTM (2005). Duplex reverse transcription-PCR followed by nested PCR
assays for detection and identification of Brazilian alphaviruses and
flaviviruses. J Clin Microbiol.

[B10] Campos J, Andrade CFS (2003). Larval susceptibility of Aedes aegypti and Culex
quinquefasciatus populations to chemical insecticides. Rev Saude Publica.

[B11] Cardoso JC, Paula MB, Fernandes A, Santos E, Almeida MAB, Fonseca DF, Sallum MAM (2010). Novos registros e potencial epidemiológico de algumas
espécies de mosquitos (Diptera, Culicidae) no estado do Rio Grande do
Sul. Rev Soc Bras Med Trop.

[B12] Carvalho-Leandro D, Ribeiro ALM, Rodrigues JSV, Albuquerque CMR, Acel AM, Santos FAL, Leite DP, Myiazaki RD (2010). Temporal distribution of Aedes aegypti Linnaeus
(Diptera, Culicidae) in a Hospital in Cuiabá, state of Mato Grosso,
Brazil. Rev Bras Entomol.

[B13] Chow VTK, Chan YC, Yong R, Lee KM, Lim LK, Chung YK, Lam-Phua SG, Tam BT (1998). Monitoring of dengue viruses in field-caught Aedes
aegypti and Aedes albopictus mosquitoes by a type-specific polymerase chain
reaction and cycle sequencing. Am J Trop Med Hyg.

[B14] Coffey LL, Forrester N, Tsetsarkin K, Vasilakis N, Weaver SC (2013). Factors shaping the adaptive landscape for arboviruses:
implications for the emergence of disease. Future Microbiol.

[B15] Cruz LCTA, Serra OP, Santos FAL, Ribeiro ALM, Dezengrini-Slhessarenko R, Santos MA (2015). Natural transovarial transmission of dengue virus 4 in
Aedes aegypti from Cuiabá, Mato Grosso, Brazil. Rev Soc Bras Med Trop.

[B16] Souza-Lopes O, Sacchetta LA, Francy DB, Jakob WL, Calisher CH (1981). Emergence of a new arbovirus disease in Brazil. III.
Isolation of Rocio virus from Psorophora ferox (Humboldt, 1819). Am J Epidemiol.

[B17] Estep LK, McClure CJW, Burkett-Cadena ND, Hassan HK, Hicks TL, Unnasch TR, Hill GE (2011). A multi-year study of mosquito feeding patterns on avian
hosts in a southeastern focus of eastern equine encephalitis virus. Am J Trop Med Hyg.

[B18] Figueiredo LTM (2000). The Brazilian flaviviruses. Microbes Infect.

[B19] Figueiredo MLG, Gomes AC, Amarilla AA, Leandro AS, Orrico AS, Araujo RF, Castro JSM, Durigon EL, Aquino VH, Figueiredo LTM (2010). Mosquitoes infected with dengue viruses in
Brazil. Virol J.

[B20] Forattini OP (2002). Culicidologia médica: identificação, biologia,
epidemiologia.

[B21] Formenti L (2015). Casos de Chikungunya no Brasil aumentam 65% em um mês e
meio.

[B22] Forrester NL, Coffey LL, Weaver SC (2014). Arboviral bottlenecks and challenges to maintaining
diversity and fitness during mosquito transmission. Viruses.

[B23] Guedes DRD (2012). Análise da competência vetorial para o vírus dengue em
populações naturais de Aedes aegypti e Aedes albopictus de Pernambuco.

[B24] Heinen LBS, Zuchi N, Cardoso BF, Santos FAL, Santos MAM, Nogueira ML, Dezengrini-Slhessarenko R (2015a). Dengue outbreak in Mato Grosso, West-Central Brazil in
2011-2012. Rev Inst Med Trop Sao Paulo.

[B25] Heinen LBS, Zuchi N, Serra OP, Cardoso BF, Gondim BHF, Santos MAM, Souto FJS, Paula DAJ, Dutra V, Dezengrini-Slhessarenko R (2015b). Saint Louis encephalitis virus in Mato Grosso,
central-western Brazil. Rev Inst Med Trop Sao Paulo.

[B26] Hoch AL, Pinheiro FP, Roberts DR, Gomes MLC (1987). Laboratory transmission of Oropouche virus by Culex
quinquefasciatus Say. Bull Pan Am Health Organ.

[B27] IBGE - Instituto Brasileiro de Geografia e Estatística (2011). Estado de Mato Grosso.

[B28] Iversson LB, Rosa APAT, Rodrigues SG, Rosa MDB (1990). Human disease caused by Venezuelan equine encephalitis subtype
IF in Ribeira Valley, São Paulo, Brazil.

[B29] Long KC, Ziegler SA, Thangamani S, Hausser NL, Kochel TJ, Higgs S, Tesh RB (2011). Experimental transmission of Mayaro virus by Aedes
aegypti. Am J Trop Med Hyg.

[B30] Luo Q (1993). A study on transmission of dengue virus by Culex
fatigans. Zhonghua Liu Xing Bing Xue Za Zhi.

[B31] Melo RM, Cavalcanti RC, Villalobos EMC, Cunha EMS, Lara MCCSH, Aguiar DM (2012). Ocorrência de equídeos soropositivos para os vírus das
encefalomielites e anemia infecciosa no estado de Mato Grosso. Arq Inst Biol.

[B32] Mondini A, Cardeal ILS, Lazaro E, Nunes SH, Moreira CC, Rahal P, Maia IL, Franco C, Góngora DVN, Góngora-Rúbio F, Cabrera EMS, Figueiredo LTM, Fonseca FG, Bronzoni RVM, Chiaravalloti F, Nogueira ML (2007). Saint Louis encephalitis virus, Brazil. Emerg Infect Dis.

[B33] Mourão MPG, Bastos MS, Figueiredo RP, Gimaque JBL, Galusso ES, Kramer VM, Oliveira CMC, Naveca FG, Figueiredo LTM (2012). Mayaro fever in the city of Manaus, Brazil,
2007-2008. Vector Borne Zoonotic Dis.

[B34] Navarro-Silva MA, Barbosa AA, Calado D (2004). Atividade de Mansonia spp em fragmento florestal na área
urbana de Curitiba, Paraná, Brasil. Rev Bras Zool.

[B35] Ng TF, Willner DL, Lim YW, Schmieder R, Chau B, Nilsson C, Al E (2011). Broad surveys of DNA viral diversity obtained through
viral metagenomics of mosquitoes. PLoS ONE.

[B36] Nogueira RMR, Eppinghaus ALF (2011). Dengue virus type 4 arrives in the state of Rio de
Janeiro: a challenge for epidemiological surveillance and control. Mem Inst Oswaldo Cruz.

[B37] Pauvolid-Corrêa A (2008). Estudos sobre arbovírus em populações de equinos e artrópodes
na sub-região da Nhecolândia no Pantanal de Mato Grosso do Sul.

[B38] Pauvolid-Corrêa A, Campos C, Juliano R, Velez J, Nogueira RMR, Komar N (2014). Serological evidence of widespread circulation of West
Nile virus and other flaviviruses in equines of the Pantanal,
Brazil. PLoS Negl Trop Dis.

[B39] Pinheiro FP, Leduc JW, Monath TP (1998). Mayaro fever. The arboviruses: epidemiology and ecology.

[B40] Powers AM, Aguilar LJC, Brault AC, Meakins AMTA, Watts D, Russel KL, Olson J, Vasconcelos PFC, Rosa AT, Weaver SC, Tesh RB (2006). Genetic relationships among Mayaro and Una viruses
suggest distinct patterns of transmission. Am J Trop Med Hyg.

[B41] Powers AM, Brault AC, Shirako Y, Strauss EG, Kang W, Strauss JH, Weaver SC (2001). Evolutionary relationships and systematics of the
alphaviruses. J Virol.

[B42] Regis L, Souza WV, Furtado AF, Fonseca CD, Silveira JC, Ribeiro PJ, Melo-Santos MAV, Carvalho MS, Monteiro AMV (2009). An entomological surveillance system based on open
spatial information for participative dengue control. An Acad Bras Cienc.

[B43] Rocco IM, Santos CLS, Bisordi I, Petrella SNCM, Pereira LE, Souza RP, Coimbra TLM, Bessa TAF, Oshiro FM, Lima LBQ, Cerroni MP, Marti AT, Barbosa VM, Katz G, Suzuki A (2005). St. Louis encephalitis virus: first isolation from a
human in São Paulo state, Brasil. Rev Inst Med Trop Sao Paulo.

[B44] Rodrigues SG, Oliva OP, Araujo FAA, Martins LC, Chiang JO, Henriques DF, Silva EVP, Rodrigues DSG, Prazeres ASC, Tavares J, Vasconcelos PFC (2010). Epidemiology of Saint Louis encephalitis virus in the
Brazilian Amazon Region and in the state of Mato Grosso do Sul, Brazil: elevated
prevalence of antibodies in horses. Rev Pan-Amaz Saude.

[B45] Schatzmayr HG (2001). Viroses emergentes e reemergentes. Cad Saude Publica.

[B46] Segura MNO, Castro FC (2007). Atlas de culicídeos na Amazônia brasileira: caracterìsticas
específicas de insetos hematófagos da família Culicidae.

[B47] SES-MT - Secretaria Estadual de Saúde do Mato Grosso (2014). Saúde do estado divulga dados da dengue de 2014 e fechamento do
ano de 2013.

[B48] Shope RE, Causey OR, Andrade AHP, Theiler M (1964). The Venezuelan equine encephalomyelitis complex of group
A arthropod-borne viruses, including Mucambo and Pixuna from the Amazon Region of
Brazil. Am J Trop Med Hyg.

[B49] Silva JR, Romeiro MF, Souza WM, Munhoz TD, Borges GP, Soares OAB, Campos CHC, Machado RZ, Silva MLCR, Faria JLM, Chávez JH, Figueiredo LTM (2014). A Saint Louis encephalitis and Rocio virus serosurvey in
Brazilian horses. Rev Soc Bras Med Trop.

[B50] Smith JL, Fonseca DM (2004). Rapid assays for identification of members of the Culex
(Culex) pipiens complex, their hybrids and other sibling species (Diptera
Culicidae). Am J Trop Med Hyg.

[B51] Svoboda WK, Martins LC, Malanski LS, Shiozawa MM, Spohr KAH, Hilst CLS, Aguiar LM, Ludwig G, Passos FC, Silva LR, Headley SA, Navarro IT (2014). Serological evidence for Saint Louis encephalitis virus
in free-ranging New World monkeys and horses within the upper Paraná River Basin
region, southern Brazil. Rev Soc Bras Med Trop.

[B52] Urdaneta L, Herrera F, Pernalete M, Zoghbi N, Rubio-Palis Y, Barrios R, Rivero J, Comach G, Jiménez M, Salcedo M (2005). Detection of dengue viruses in field-caught Aedes
aegypti (Diptera: Culicidae) in Maracay, Aragua state, Venezuela, by type-specific
polymerase chain reaction. Infect Genet Evol.

[B53] Vazeille M, Mousson L, Martin E, Failloux AB (2010). Orally co-infected Aedes albopictus from La Reunion
Island, Indian Ocean, can deliver both dengue and Chikungunya infectious viral
particles in their saliva. PLoS Negl Trop Dis.

[B54] Vega-Rúa A, Zouache K, Girod R, Failloux AB, Lourenço-de-Oliveira R (2014). High vector competence of Aedes aegypti and Aedes
albopictus from ten American countries as a crucial factor of the spread of
Chikungunya. J Virol.

[B55] Vieira CJSP, Silva DJF, Barreto ES, Siqueira CEH, Colombo TE, Ozanic K, Schmidt DJ, Drumond BP, Mondini A, Nogueira ML, Bronzoni RVM (2015). Detection of Mayaro virus infection during a dengue
outbreak in Mato Grosso, Brazil. Acta Trop.

[B56] WHO - World Health Organization (2014). West Nile virus - Brazil.

[B57] Zeidler JD, Acosta POA, Barrêto PP, Cordeiro JS (2008). Virus dengue em larvas de Aedes aegypti e sua dinâmica
de infestação, Roraima, Brasil. Rev Saude Publica.

[B58] Zuchi N, Heinen LBS, Santos MAM, Pereira FC, Slhessarenko RD (2014). Molecular detection of Mayaro virus during a dengue
outbreak in the state of Mato Grosso, central-west Brazil. Mem Inst Oswaldo Cruz.

